# Altered cortical Cytoarchitecture in the *Fmr1* knockout mouse

**DOI:** 10.1186/s13041-019-0478-8

**Published:** 2019-06-14

**Authors:** Frankie H. F. Lee, Terence K. Y. Lai, Ping Su, Fang Liu

**Affiliations:** 1 Campbell Family Mental Health Research Institute, Centre for Addiction and Mental Health, Toronto, Ontario M5T 1R8 Canada; 2 Department of Physiology, University of Toronto, Toronto, Ontario M5T 1R8 Canada; 3 Department of Psychiatry, University of Toronto, Toronto, Ontario M5T 1R8 Canada

**Keywords:** Fragile X syndrome, *Fmr1* KO mice, Cortical architecture, Astrocytes

## Abstract

Fragile X syndrome (FXS) is a neurodevelopmental disorder caused by silencing of the *FMR1* gene and subsequent loss of its protein product, fragile X retardation protein (FMRP). One of the most robust neuropathological findings in post-mortem human FXS and *Fmr1* KO mice is the abnormal increase in dendritic spine densities, with the majority of spines showing an elongated immature morphology. However, the exact mechanisms of how FMRP can regulate dendritic spine development are still unclear. Abnormal dendritic spines can result from disturbances of multiple factors during neurodevelopment, such as alterations in neuron numbers, position and glial cells. In this study, we undertook a comprehensive histological analysis of the cerebral cortex in *Fmr1* KO mice. They displayed significantly fewer neuron and PV-interneuron numbers, along with altered cortical lamination patterns. In terms of glial cells, *Fmr1* KO mice exhibited an increase in Olig2-oligodendrocytes, which corresponded to the abnormally higher myelin expression in the corpus callosum. Iba1-microglia were significantly reduced but GFAP-astrocyte numbers and intensity were elevated. Using primary astrocytes derived from KO mice, we further demonstrated the presence of astrogliosis characterized by an increase in GFAP expression and astrocyte hypertrophy. Our findings provide important information on the cortical architecture of *Fmr1* KO mice, and insights towards possible mechanisms associated with FXS.

**Electronic supplementary material:**

The online version of this article (10.1186/s13041-019-0478-8) contains supplementary material, which is available to authorized users.

## Introduction

Fragile X syndrome (FXS) is the most common inherited form of intellectual disability and one of the leading genetic causes of autism spectrum disorder (ASD), affecting approximately 1 in 4000 males and 1 in 6000 females [1–3]. It is characterized by a wide spectrum of clinical symptoms, including mild to severe intellectual disability, susceptibility to seizures, hyperactivity, hypersensitivity to sensory stimuli, autistic behaviors such as social anxiety and attention deficits, macroorchidism and abnormal facial features (Reviewed in [3]). FXS is a neurodevelopmental disorder caused by a CGG repeat expansion in the X-linked fragile X mental retardation 1 (*FMR1*) gene, which results in the transcriptional silencing of *FMR1*, and subsequent reduction of its protein product, fragile X retardation protein (FMRP) [4, 5].

*FMR1* and FMRP are ubiquitously expressed in the mammalian CNS, beginning in early embryogenesis and persisting throughout development into adulthood [6]. At the cellular level, they are detected in different cell populations from proliferating cells of the developing brain, and later within pyramidal neurons, to GABAergic interneurons and glial cells of microglia, oligodendrocytes and astrocytes [6–11]. Functionally, FMRP regulates mRNA expression by binding and then suppressing the translation of its target mRNAs [3]. Genome-wide microarrays and high-throughput sequencing studies have identified more than 800 mRNA targets of FMRP, many of which are linked to neurodevelopmental processes including neurite growth, spine development, synaptic function and neuronal signaling [12–14]. The broad expression of FMRP in multiple cell types and brain regions, together with the vast number of interacting mRNA targets, suggest that it is immensely important for brain development and maturation.

Animal models are crucial in understanding the biological functions of genetic mutations. The *Fmr1* knockout (KO) mouse, containing the loss of a functional FMRP protein, has been well-established in displaying behavioral abnormalities reminiscent of human FXS traits [15, 16]. More importantly, many studies have since been using this model as a tool to investigate the pathophysiological mechanisms underlying FXS. One of the most robust neuropathological findings in post-mortem human FXS and *Fmr1* KO mice is the abnormal increase in dendritic spine densities, with the majority of spines showing an elongated immature morphology [17–19]. However, evidence of other histological defects associated with FXS are still lacking, and inconsistent results have been described on neurotransmission properties, such as NMDA and AMPA receptor expression/function [3].

Dendritic spine development is a complicated process beginning with synaptogenesis in early childhood, synapse elimination/pruning in adolescence and spine maintenance in adulthood [20]. Proper development and maturation requires strict spatial and temporal regulation, involving multiple factors at each stage. Therefore, alterations in spine number and/or morphology could emerge from secondary effects or compensatory responses of any dysfunctional events occurring during that period. For example, neuron and interneuron numbers, cortical lamination pattern, and axonal connections and myelination have all been shown to affect spinogenesis [21–23]. Besides, recent evidence has directed microglia and astrocytes as key players in modulating synaptic formation and pruning via distinct pathways. For example, microglia and astrocytes mediate synapse elimination by direct engulfment of synapses through the complement system and MEGF10/MERTK respectively [24, 25]. Moreover, alterations in cytokine production and astrocyte-specific glutamate transporters can have pronounced effects on dendritic spines [26–28].

Despite extensive research focusing on the functional role of FMRP in FXS, how it regulates spine development remains unclear. In this study, we undertook a comprehensive histological analysis of the cerebral cortex in the *Fmr1* KO mice. They displayed reductions in cortical neuron and PV-interneuron numbers, along with altered cortical lamination patterns. In terms of glial cells, KO mutants exhibited distinct changes in Olig2-oligodendrocyte, Iba-1 microglia and GFAP-astrocyte numbers. Using primary astrocytes from *Fmr1* KO mice, we further demonstrated the presence of astrogliosis characterized by the significant increase in GFAP expression and astrocyte hypertrophy. Our findings provide important information on the cortical architecture of *Fmr1* KO mice, and insights towards possible mechanisms that could be responsible for the increase in spine densities associated with FXS.

## Results

### Fewer neuron and parvalbumin (PV)-interneuron numbers in the *Fmr1* KO mouse cortex

**Fig. 1:**
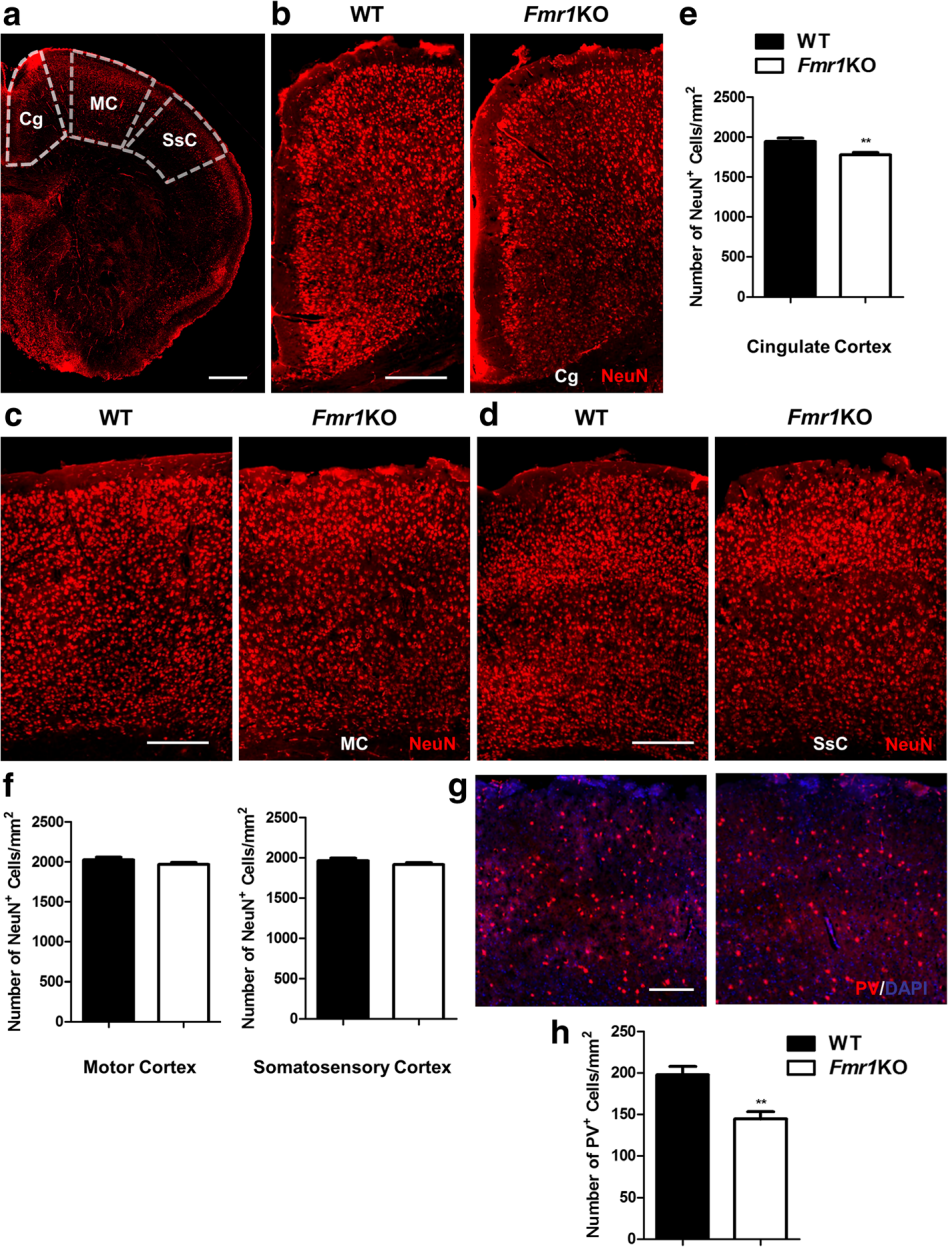
Fewer neuron and parvalbumin (PV)-interneuron numbers in the *Fmr1* KO mouse cortex. **a** Representative fluorescent images of NeuN-immunostained cells outlining the regions of interest across the neocortex, which includes the cingulate, motor and somatosensory cortex in WT and *Fmr1* KO mice. Scale Bar: 300 μm. **b-d** Higher magnification images for each region are correspondingly shown. Scale Bar: 100 μm. **e, f** The number of NeuN-positive cells in the cingulate cortex of KO mice is significantly lower than WT, but not the motor or somatosensory regions (*n* = 24 per group). **g, h** Immunolabeling of parvalbumin (PV) shows that *Fmr1* KO mice also have less PV-interneurons in the neocortex when compared to WT (*n* = 20–24 per group). Scale Bar: 100 μm. Data are presented as mean ± SEM. **p < 0.01. Cg: cingulate cortex, MC: motor cortex, SsC: somatosensory cortex

FMRP is strongly expressed in both pyramidal and GABAergic neurons from embryonic development to adulthood [6, 11], indicating that it is likely to play a role in their maturation and functions. First, we examined the number of neurons across the neocortex in the cingulate, motor and somatosensory regions of *Fmr1* KO mice (Fig. 1a). NeuN-immunolabeling of neurons revealed a significant reduction within the cingulate cortex of KO mice (WT: 1945 ± 188 cells per mm^2^; KO: 1777 ± 144 cells per mm^2^) but not the motor or somatosensory regions (Fig. 1b-f). NeuN labels both pyramidal neurons and interneurons in the cortex [29], and since interneurons only comprise of approximately 20–30% of all cortical neurons [30], there is a possibility that the results may mask any differences in interneuron numbers. Hence we immunolabeled parvalbumin (PV), which is the most common interneuron subtype responsible for fast-spiking activity [31]. Total number PV-cells were significantly fewer in the neocortex of KO mutants when compared to WT (WT: 198 ± 45 cells per mm^2^; KO: 144 ± 44 cells per mm^2^) (Fig. 1g, h).

### *Fmr1* KO mice show altered cortical lamination

**Fig. 2:**
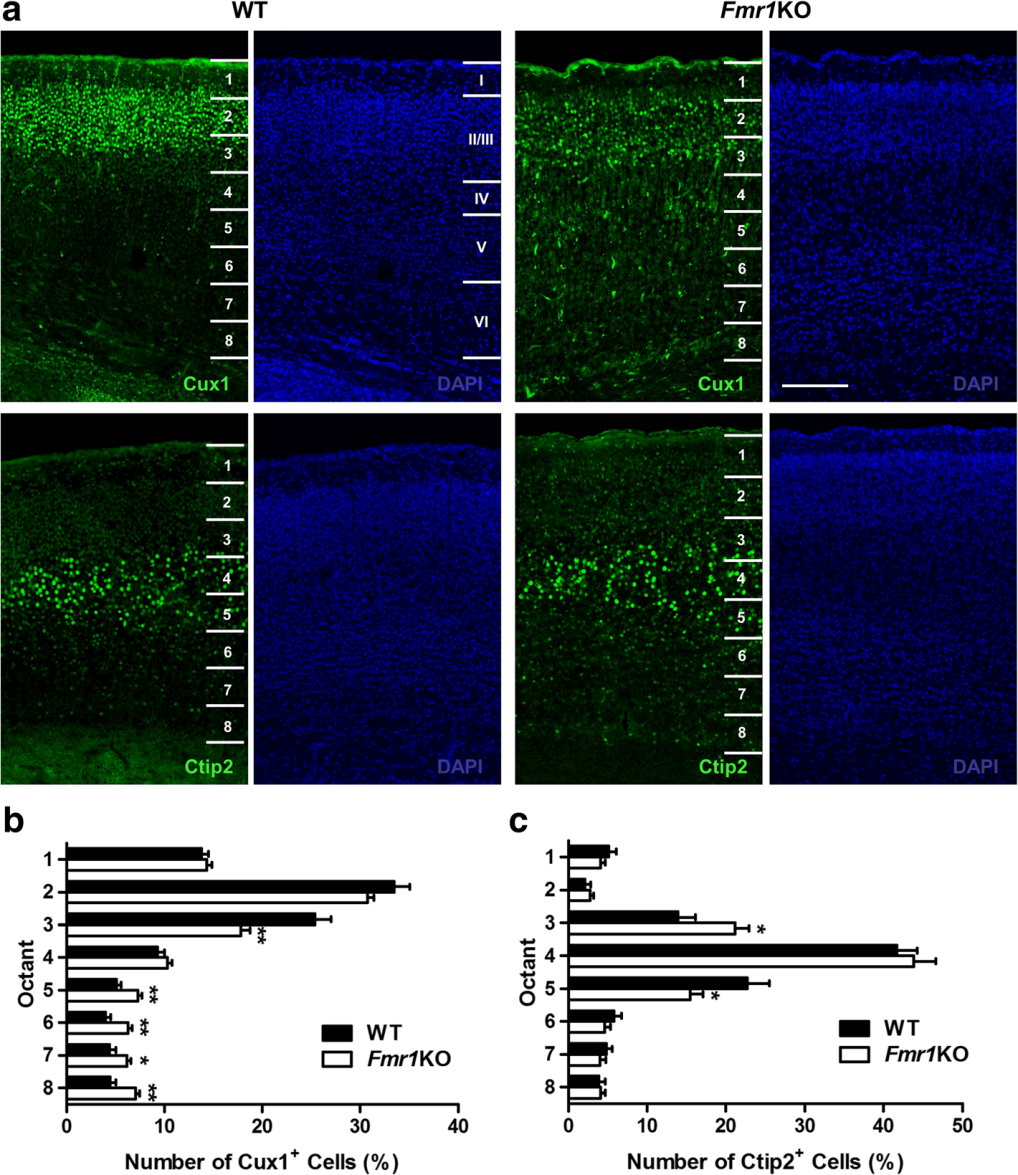
Alterations of cortical neuronal distribution in *Fmr1* KO mice. **a** Immunohistochemistry with Cux1, a layer II/III-specific protein marker, and Ctip2, a layer V-marker is performed in WT and *Fmr1* KO mice. Regions of interest (ROIs) are positioned over the neocortex with each ROI further subdivided into eight equal bins spanning the pia to the inner border of the cortex, to assess neuron distribution in different cortical layers. The number of Cux1^+^ and Ctip2^+^ cells are counted in each bin and expressed as a percentage total numbers. Scale Bar: 100 μm. **b** The proportion of Cux1-labeled cells is significantly lower in bin 3 near superficial layers II/III, along with a corresponding increase in deeper layers of bins 5, 6, 7 and 8 for KO mice relative to WT (*n* = 24–32 per group). **c** As for Ctip2, there is a larger proportion of cells positioned in bin 3 but less in deeper layer bin 5 of KO mutants when compared to controls (*n* = 30–32 per group). Data are presented as mean ± SEM. *p < 0.05, **p < 0.01

Next, we analyzed the effects of FMRP knockout on cortical neuronal positioning by performing immunohistochemistry with Cux1, a layer II/III-specific protein marker, and Ctip2, a layer V-marker [32]. To quantify the positions of Cux1- and Ctip2-labeled cells, a rectangular region of interest spanning the whole cortical layers I-VI was positioned over the neocortical region of fluorescent-labeled cells, and further subdivided into eight equal bins (Fig. 2a) [33]. The percentage of Cux1-labeled cells was significantly fewer in bin 3 near superficial layers II/III for KO mice relative to WT, but displayed a corresponding increase in deeper layers of bins 5, 6, 7 and 8 (Fig. 2b). These results indicate that neurons destined for superficial layers did not reach their correct location in the KO mutants. As for Ctip2, there was a larger proportion of cells positioned in bin 3 for KO groups, but a lower proportion was observed in deeper layers of bin 5 (Fig. 2c). Together, our data suggest an aberrant localization of cortical neurons in the *Fmr1* KO mice.

### Distinct changes in glial cell numbers of *Fmr1* KO mice

**Fig. 3:**
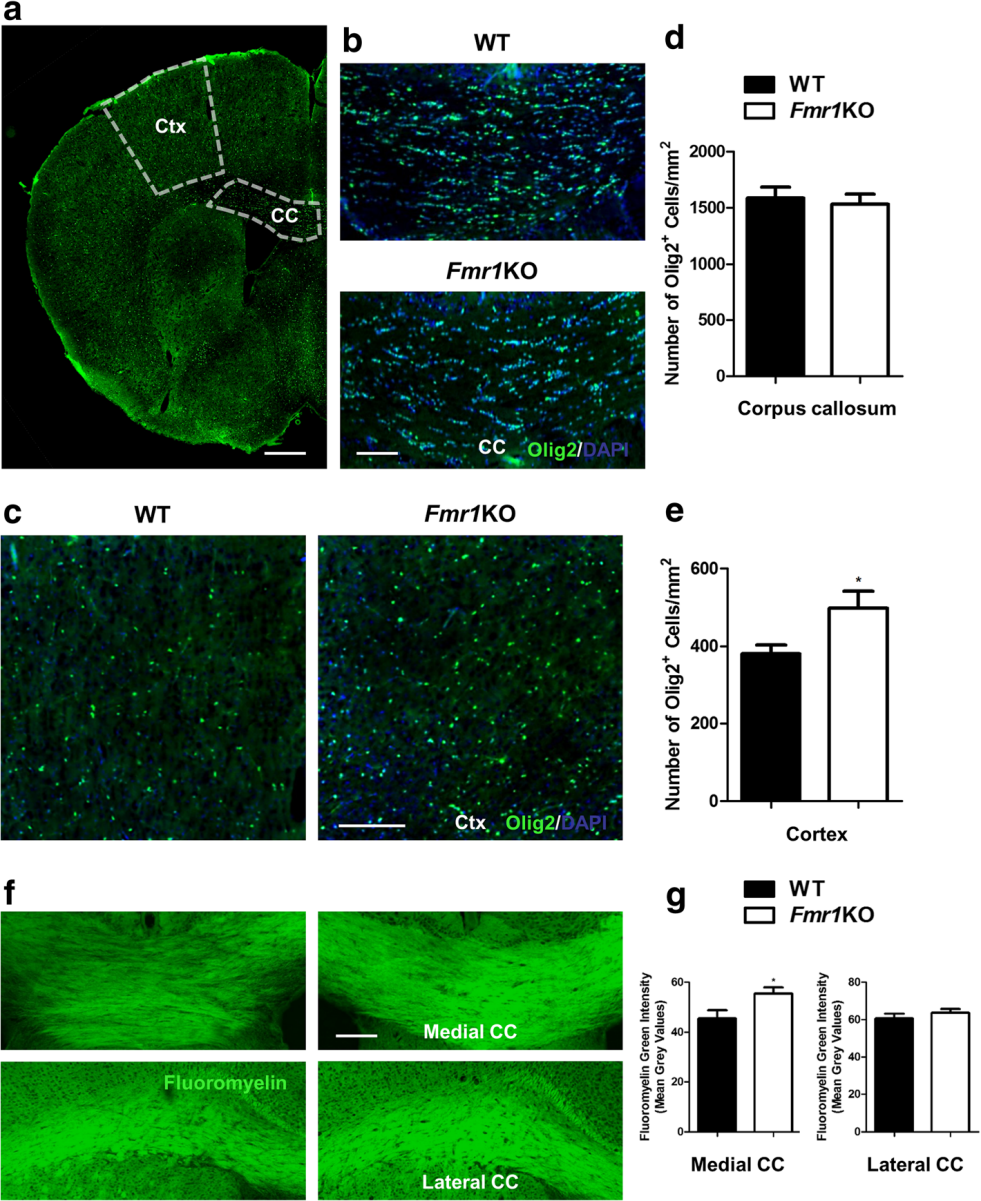
*Fmr1* KO mice show significantly more oligodendrocytes and enhanced myelination in the cortex**. a** Representative fluorescent images of Olig2 immunostaining of WT and *Fmr1* KO brain coronal sections delineating the cortex and corpus callosum. Scale Bar: 300 μm. **b, c** Higher magnification images of corpus callosum and cortex are shown. **d, e** Compared to WT, KO mice have significantly more Olig2-positive pre-myelinating oligodendrocytes in the cortex but no difference is observed in the corpus callosum (*n* = 20–24 per group). **f** Fluoromyelin green was used to label myelin tracts in the medial and lateral part of the corpus callosum. **g** Likewise, myelin intensity is increased within the medial region but not the lateral sides of KO mutants (*n* = 14–16 per group). Scale Bar: 100 μm. Data are presented as mean ± SEM. *p < 0.05. Ctx: cortex, CC: corpus callosum

There is evidence indicating the presence of FMRP in glial cells of oligodendrocytes, microglia and astrocytes during early and mid-postnatal developmental stages of brain maturation [6, 7, 10], which imply possible FMRP regulatory functions in these cell types. Hence we investigated the histological features of oligodendrocytes, microglia and astrocytes in the *Fmr1* KO mouse cortex. Immunostaining of Olig2, a marker of pre-myelinating oligodendrocytes, resulted in a significant increase in oligodendrocyte numbers within the cortex, but not the corpus callosum of KO mice when compared to WT (WT: 381 ± 110 cells per mm^2^; KO: 499 ± 190 cells per mm^2^) (Fig. 3a-e). Since the primary function of oligodendrocytes is to myelinate axons which enables faster propagation of nerve impulses in the central nervous system (CNS), we further analyzed myelin expression in the medial and lateral corpus callosum (Fig. 3f). Using fluoromyelin-green, we observed a higher fluorescent intensity of myelin only in the medial part (WT: 45.48 ± 10.95; KO: 55.48 ± 7.81). However, there was no difference within the lateral region of each hemisphere (Fig. 3g). The abnormal increase in cortical pre-myelinating oligodendrocytes may be responsible for the enhanced myelination observed in the medial corpus callosum.

**Fig. 4:**
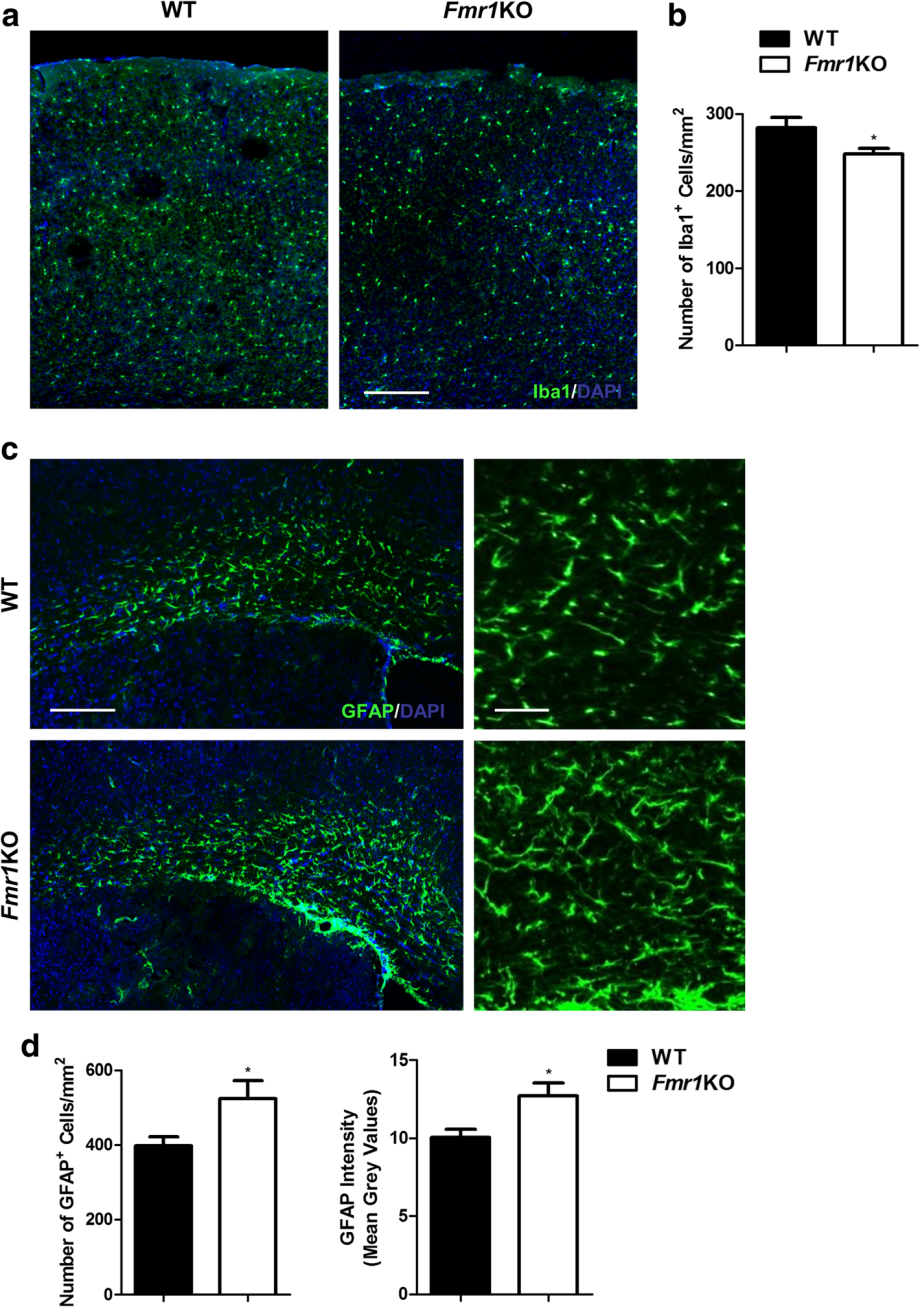
*Fmr1* KO mice have less Iba1-positive microglia but more GFAP-astrocytes**. a** WT and *Fmr1* KO mouse cortices are immunostained with Iba1 for microglia analysis. Scale Bar: 100 μm. **b** Quantification of Iba1-positive cells reveals fewer numbers in *Fmr1* KO mutants (*n* = 46–48 per group). **c** Anti-GFAP is used to label astrocytes in the corpus callosum. Scale Bar: 100 μm. Higher magnification images are shown on the right. Scale Bar: 50 μm. **d** *Fmr1* KO mutants have substantially more GFAP-astrocytes and higher GFAP expression (*n* = 14–16 per group). Data are presented as mean ± SEM. *p < 0.05

In addition, we performed immunohistochemistry with anti-Iba1, a marker of microglia and anti-GFAP, a marker of astrocytes in WT and *Fmr1* KO mice. The number of Iba1-positive microglia was significantly lower in the cortex of KO groups vs. WT (WT: 282 ± 91 cells per mm^2^; KO: 248 ± 49 cells per mm^2^) (Fig. 4a, b). In contrast, *Fmr1* KO mice exhibited more GFAP-immunoreactive astrocyte numbers (WT: 398 ± 76 cells per mm^2^; KO: 524 ± 169 cells per mm^2^), as well as higher GFAP fluorescent intensities (WT: 10.04 ± 1.68; KO: 12.73 ± 2.92) (Fig. 4c, d). It appears that FMRP deletion reduced the number of activated microglia, but promoted the state of astrogliosis. Together, our results demonstrated distinct alterations in the number of different glial cell types and in specific cortical regions.

### FMRP-depleted astrocytes exhibit a more pronounced reactive state with higher GFAP expression and hypertrophy

**Fig. 5:**
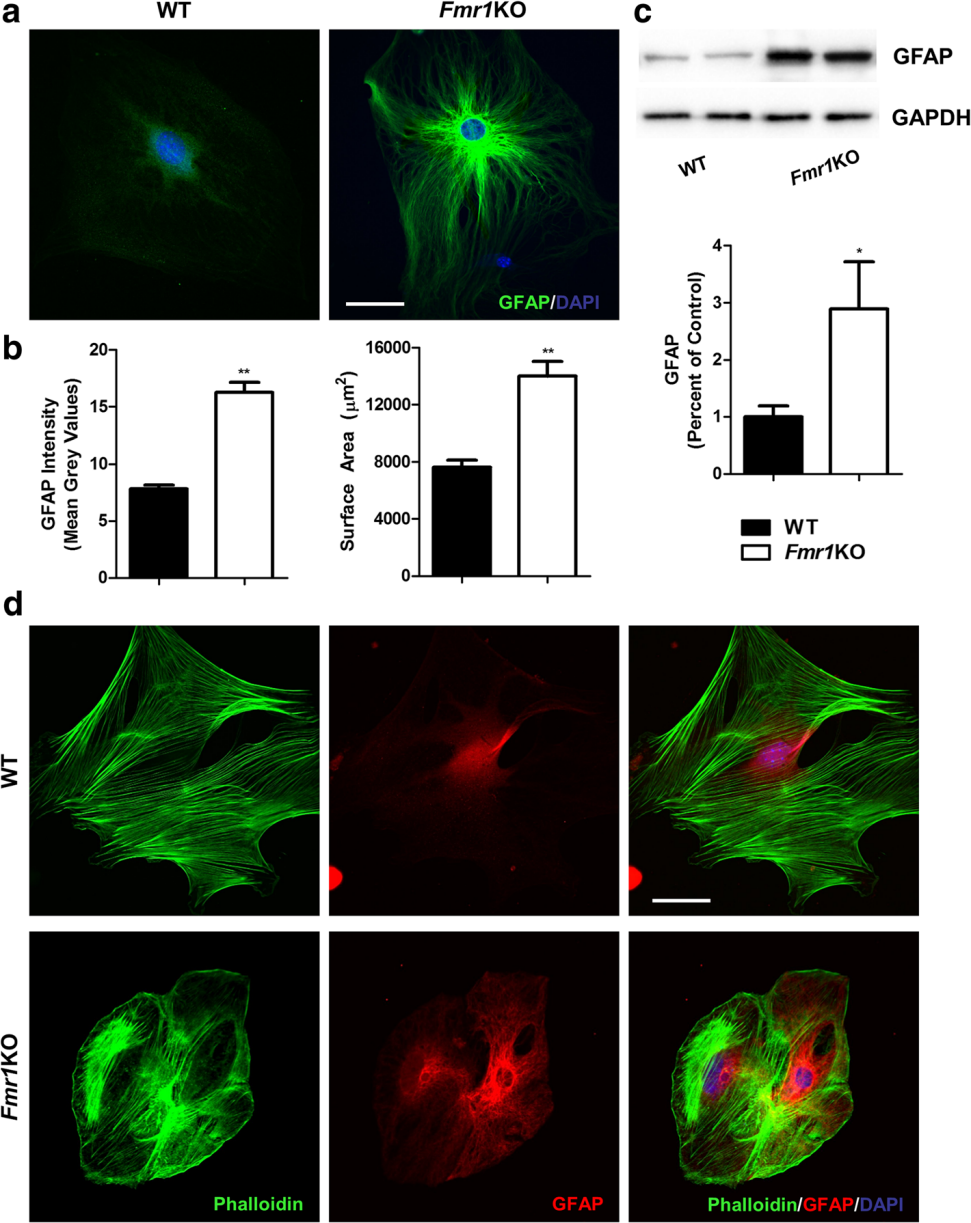
FMRP-deficient astrocytes become reactive and display actin reorganization**. a** Primary astrocyte cultures derived from WT and *Fmr1* KO neonatal brains are immunostained with GFAP. **b** KO-astrocytes have a pronounced increase in GFAP fluorescent intensity and astrocyte surface area, which are characteristics of reactive astrogliosis (*n* = 30–34 per group). **c** Western blot analysis confirms that GFAP expression is substantially higher in *Fmr1* KO astrocytes (*n* = 4 different cultures per group). **d** Representative fluorescent images showing F-actin fibres (labeled with phalloidin) in GFAP-astrocyte cultures. Normal astrocytes display a distinct well-organized evenly distributed pattern, but F-actin in FMRP-depleted astrocytes form clusters in the cytoplasmic region. Scale Bar: 20 μm. Data are presented as mean ± SEM. *p < 0.05, **p < 0.01

To further address the direct effects of FMRP ablation on astrocytes reactivity, we prepared primary astrocyte cultures from neonatal cortical tissues of WT and *Fmr1* KO brains. First, the lack of FMRP protein in astrocytes was confirmed in the KO group using immunocytochemistry and Western blot (Additional file 1: Figure S1a, b). Astrogliosis is characterized by profound molecular and morphological changes in astrocytes in response to CNS injuries and diseases. These include a marked increase in GFAP expression and morphologically, astrocytes undergo extensive hypertrophy and proliferation [34, 35]. At the individual cell level, *Fmr1* KO astrocytes displayed a profound increase in GFAP intensity (WT: 7.82 ± 1.88; KO: 16.28 ± 4.71) and astrocyte GFAP surface area (WT: 7647 ± 2834 μm^2^; KO: 14014 ± 5553 μm^2^) (Fig. 5a, b). Western blot analysis also validated the enhanced GFAP expression in astrocyte cultures of KO mutants (Fig. 5c). Actin reorganization has been shown to be a main determinant of cellular morphology, which is responsible for the morphological changes in reactive astrocytes. Indeed, evidence illustrating the loss and disassembly of F-actin fibres was found in different models of astrogliosis [36, 37]. As shown in Fig. 5d, we did not detect any loss of phalloidin-labeled F-actin fibres in *Fmr1* KO astrocytes when compared to WT. However, marked alterations in the assembly of F-actin were observed between the groups. Specifically, actin within normal astrocytes was detected to be more evenly distributed outlining the shape of each cell, but abnormal clusters were formed in the middle part of *Fmr1* KO astrocytes (Fig. 5d). These findings suggest that abolishing FMRP results in astrogliosis, which may contribute to improper synaptic pruning and aberrant spine numbers.

## Discussion

There is strong evidence indicating the diverse roles of FMRP in regulating many essential neurodevelopmental processes [3]. The most robust histopathological finding in FXS patients and *Fmr1* KO mice is the abnormal increase in immature dendritic spine numbers [17–19]. However, the mechanisms of how FMRP can modulate spine development are still unclear. Disturbances in many neurodevelopmental factors can have profound effects on dendritic spines. In this study, we examined the cortical cytoarchitecture in the *Fmr1* KO mouse cortex that could be associated with spine abnormalities. Alterations in neuronal number and positioning can affect proper spine formation. There is evidence suggesting that neuronal populations inherently respond to lower neuronal densities by increasing the number of synapses [23]. We observed a small but significant decrease in neuronal density only within the cingulate cortex of KO mutants but not the lateral regions of motor and somatosensory cortex. Consistently, Hall et al. reported that patients with FXS had reduced gray matter density in the cingulate cortex using voxel-based morphometry [38]. In the *Fmr1* KO mouse, a study found no change in gross cortical neuronal morphology or overall cortical thickness of the primary somatosensory cortex [39]. Earlier studies have documented the involvement of FMRP in embryonic neurogenesis, where embryonic *Fmr1* KO mice displayed accelerated neurogenesis, increased neuronal differentiation and enhanced accumulation of progenitors in the subventricular zone [40–42]. These newly differentiated FMRP-deficient neurons exhibit abnormal functional properties such as altered calcium responses and bursts activity [40, 43]. As a result, non-functional neurons that could not be integrated into the local circuitry are eliminated, which may explain the reduction in neuron number.

FMRP is also prominently expressed in GABAergic neuron populations, implying that it may have crucial roles in interneuron development and function [8, 11]. Indeed, using the *Fmr1* KO mouse model, many studies have illustrated defects in the inhibitory GABAergic circuitry [11, 44]. We found significantly fewer PV-interneurons in all regions across the neocortex, which was coherent with the results reported by Selby et al. [45]. Furthermore, according to Selby et al., this reduction mainly occurred in somatosensory cortical layers of II/III/IV, but not in deeper layers V and VI where PV numbers were increased [45]. These findings suggest that there could be differential histological deficits within cortical laminar organization. We noticed an aberrant cortical lamination pattern in the *Fmr1* KO mutants compared to WT. Neurons destined for deeper layers were mis-positioned to superficial layers while those for superficial remained in deeper regions. Newborn neurons are established in an ‘inside-out’ pattern with the earliest born neurons forming the deep cortical layers, followed by later born neurons that progressively migrate into more superficial layers [32, 46, 47]. Our data corroborated with previous findings of more cortical neurons mis-positioned in deeper layers of *Fmr1* KO embryonic brains, and further indicate that this effect persists into adulthood [48]. It is possible that FMRP deficiency may contribute to an accelerated neurogenesis in which more premature neurons are misdirected to deeper localization. Another explanation for neuronal mis-positioning in the cortex could be due to deficits in the migration process. Although some research have documented the role of FMRP in regulating neuronal migration [49–51], La Fata et al. reported that FMRP depletion did not affect radial migration of excitatory neurons [48]. A better understanding towards how FMRP modulates these processes associated with FXS is necessary. Nonetheless, this study provides a clearer histopathological perspective of neuron and interneuron numbers, and cortical laminar positioning in *Fmr1* KO mice, as well as reflecting the possible neurodevelopmental processes affected by FMRP depletion.

FMRP expression is not only restricted to neuronal populations, but also present in developing and mature glial cells including oligodendrocytes, microglia and astrocytes [6, 7, 10]. Oligodendrocytes are primarily responsible for myelinating axons which enables faster and more efficient propagation of nerve impulses [52]. Hence, proper myelination is essential for normal neuronal development including dendritic spines [53]. Although different studies have indicated abnormal white matter structure in FXS patients and *Fmr1* KO mice, the findings are still inconsistent [54]. We detected a pronounced increase in Olig2-positive pre-myelinating oligodendrocytes in the neocortex but not the corpus callosum of *Fmr1* KO mice. Correspondingly, myelination only within the medial part of the corpus callosum was abnormally enhanced. In support of our results, a study found that oligodendrocyte ablation could lead to an increase in synapse numbers, possibly via modification of ionotropic glutamate receptors localization and function [55]. Both diffusion tensor imaging studies in FXS patients and magnetic resonance imaging studies in *Fmr1* KO mice have shown an increase in white matter tract density [56, 57], despite others have reported reduced myelination patterns [9, 58]. A more comprehensive study from Hall et al. suggested that white matter abnormalities associated with FXS are region-specific [54]. As the developmental origins of different brain regions are distinct, the functional role of FMRP on oligodendrocyte development and myelination may also differ. Moreover, there is evidence illustrating that FMRP can bind and regulate the translation of myelin basic protein (MBP) and CNPase (a myelin-associated enzyme) [10, 13]. However, it remains unclear how FMRP modulate the different aspects of oligodendrocyte development, for instance the differentiation of oligodendrocyte precursor cells to mature oligodendrocytes, or apoptosis of oligodendrocytes. A more detail investigation on these events can provide important clues on the relationship between FMRP and white matter function associated with FXS.

Accumulating evidence strongly indicates that both microglia and astrocytes are involved in many neurodevelopmental processes, thus are immensely crucial in maintaining normal brain functions [59–61]. With regards to FXS, microglia can directly mediate dendritic spine elimination via the complement system where excessive synapses are tagged with complement proteins (C1q and C3) and consequently engulfed by microglial cells expressing complement receptors [24, 62, 63]. There is clinical evidence suggesting excessive microglial activation in multiple brains regions of patients with ASD [64, 65], but other studies did not detect any microglial activation or altered cytokine production in the *Fmr1* KO mice [66, 67]. In our study, we found a slight reduction in the number of Iba1-positive cells across the neocortex of *Fmr1* KO mutants, further justifying the absence of microglial activation. It is likely that the reduction in microglia leads to less synapse elimination mediated by the complement pathway [68], and other mediators such as IL-33 and CX3CR1s [69].

The importance of FMRP in astrocytes was validated by a recent study that showed astrocyte-specific FMRP knockout in mice resulted in increased spine density and behaviors reminiscent of FXS [70]. Astrocytes are a major source of complement C3, and therefore could participate in removing synapses by secreting C3 [68]. Similar to microglia, a study by Chung et al. described a direct phagocytic role of astrocytes on CNS synapses through phagocytic receptors, such as MEGF10 and MERTK [25]. In addition, astrocytes can also contribute to synaptic elimination via inositol 1,4,5-triphosphate receptor type 2 (IP3R2) and *P2ry1* dependent signaling [71]. Here, we observed that *Fmr1* KO mice exhibited prominent GFAP expression, while in vitro astrocyte cultures displayed hypertrophy and increased GFAP levels, all of which are hallmark features of reactive astrogliosis. In support of our findings, separate studies have provided evidence for astrocytic activation in the cerebellum of *Fmr1* KO mouse [66, 67] and in patients with autism [65, 72]. Zamanian et al. conducted an extensive analysis on the genomic profile of reactive astrocytes, where they identified numerous genes of different signaling pathways were significantly induced [73]. FMRP-deficient reactive astrocytes are likely to exhibit similar alterations in gene expression that could contribute to the abnormal increase in dendritic spines. For instance, changes in genes of the complement pathway can affect the process of synapse tagging and removal [68]; and induction of cytokine production such as IL-6 is evidently linked to dendritic spine abnormalities as well as behaviors relevant to autism [26, 27]. Another possible mechanism can be attributed to the reduced expression of the astrocyte-specific glutamate transporter GLT1 in FMRP-deleted astrocytes, as selective re-expression of FMRP rescued abnormal spine morphology [28]. The detail mechanisms of how reactive astrogliosis in *Fmr1* KO can cause an abnormal increase in dendritic spines would require more research on deciphering the downstream pathways.

In conclusion, we found that *Fmr1* KO mice displayed histological deficits of fewer neuron and PV-interneuron numbers, altered cortical lamination, more Olig2-oligodendrocytes with enhanced myelination expression, less Iba1-microglia and elevated GFAP expression and astrocyte numbers. Moreover, using primary astrocyte cultures from *Fmr1* KO mouse cortex, we demonstrated the presence of astrogliosis characterized by pronounced GFAP expression and hypertrophy at the cellular level. These histopathological defects are strong indicators of perturbed neurodevelopmental processes that could either directly or indirectly contribute to the abnormal elevated spine numbers associated with FXS. Further studies on neurodevelopmental pathways such as neurogenesis, neuronal migration and glial development can address this question. More importantly, our results provide a starting point for future research in investigating at the specific pathways involved in dendritic spine formation and pruning, which could ultimately lead to novel treatments in FXS.

## Methods

### Mice

*Fmr1* KO mice on a C57/BL6J background were purchased from The Jackson Laboratory (B6.129P2-*Fmr1*^tm1Cgr^/J, Stock No: 003025), and bred at the Centre for Addiction and Mental Health (CAMH) (Toronto, Canada). Mice of approximately 4 weeks old were used for all experiments. Control groups were conducted with WT mice. All mouse protocols were approved by the CAMH Animal Care Committee and methods were carried out in accordance with the approved guidelines.

### Primary astrocyte culture preparation

Cortical tissues from postnatal day 1–3 (P1–3) mouse brains were dissected out, incubated with 0.25% trypsin for 15 min at 37 °C, and dissociated by mechanical trituration. Cells were plated on T25 cell culture flasks, and grown in DMEM with 10% fetal bovine serum (FBS), 100 U/ml penicillin and 100 μg/ml streptomycin in an incubator (37 °C, 5% CO_2_) until astrocyte monolayers were confluent. Half of the DMEM was replaced every 3–4 days. To remove microglia and oligodendrocytes from mixed glial cultures, flasks were shaken for 24 h at 200 rpm on an orbital shaker. Afterwards, versene solution and 2.5% trypsin were added to disaggregate astrocytes completely. Purified astrocytes were resuspended in DMEM/10% FBS and plated on either glass coverslips or 60 mm culture dishes, and grown until approximately 50% confluency for immunocytochemistry or fully confluent for Western blot experiments. The purity of astrocytes was confirmed with GFAP/DAPI labeling.

### Immunofluorescence

#### Immunohistochemistry

Adult mouse brains (approximately 8 weeks of age) were harvested, fixed in 4% paraformaldehyde (PFA) overnight, cryoprotected in 30% sucrose and frozen at − 80 °C before further processing. 20 μm-thickness frozen coronal sections were cut using a microtome cryostat system (Leica CM3000). Free floating sections were initially blocked in 5% fetal bovine serum, 1% Triton X-100, 0.5% Tween 20 and 1% skim milk in 0.1 M PBS for 2 h at room temperature to reduce non-specific binding. This was followed by incubation with primary antibodies overnight at 4 °C and secondary antibodies for 2 h in blocking solution at room temperature. The primary antibodies used in this study include: anti-NeuN (1:200, Millipore MAB377, Etobicoke, Canada), anti-Parvalbumin (1:200, Sigma-Aldrich P3088, Oakville, Canada), anti-CDP (M-222) (Cux1) (1:200, Santa Cruz Biotechnology sc-13,024, Dallas, TX, USA), anti-Ctip2 (1:200, Abcam ab28448, Cambridge, MS, USA), anti-Olig2 (1:200, Abcam ab109186), anti-Iba1 (1:500, Wako 019–19,741, Richmond, VA, USA) and anti-GFAP (1:200, Dako Z0334, Glostrup, Denmark). Alexa 488- or 594-conjugated secondary antibodies (1:200; Thermo Fisher Scientific, Burlington, Canada) in blocking solution were used for detection of primary antibodies. Staining of myelin and nuclei was performed with Fluoromyelin green and DAPI (Thermo Fisher Scientific) respectively.

#### Immunocytochemistry

Astrocyte cultures were fixed in 4% PFA/4% sucrose, permeabilized with 0.1 M PBS containing 0.1% Triton X-100 for 15 min, and blocked for 1 h with 1% bovine serum albumin in PBS at room temperature. Likewise, they were incubated with primary antibodies overnight at 4 °C and secondary antibodies for 1 h at room temperature. Primary antibodies used for this part include anti-GFAP (1:500; Dako Z0334) and anti-Fmrp (1:200, Abcam ab17722). Alexa 488- phalloidin (Thermo Fisher Scientific A12379) and DAPI were also applied to label F-actin and nuclei respectively.

### Immunofluorescence analysis

Fluorescent images of brain sections and astrocyte cultures were captured using a confocal microscope (Olympus FluoView FV1200) at either 10× or 60× magnification. For immunohistochemistry, slices chosen for analyses were anatomically-matched between comparing groups, and included samples from rostral to caudal regions. All images were converted to grey-scale and normalized to background staining. Fluorescently-labeled cells were quantified in specifically-defined regions of the cortex using the ITCN plugin for ImageJ (https://imagej.nih.gov/ij/), where fixed parameters of cell width and threshold are pre-set such that only cells reaching the minimum signal will be counted. For Cux1 and Ctip2, regions of interest (ROIs) were positioned over cortical regions with each ROI further subdivided into eight equal bins from the pia to the inner border of the cortex, to assess neuron distribution across the layers of the cortex (Fig. 2a) [33]. The distribution was expressed as a percentage of the numbers of labeled-cells in each bin divided by the total numbers within each ROI [33]. In addition, fluorescent signal intensities of myelin staining and GFAP immunoreactivity were measured by mean grey values (ImageJ). Similarly with immunocytochemistry on astrocytes, the cell perimeter was first outlined and mean grey values of GFAP fluorescence, as well as surface area were determined as previously described. All image-capturing and threshold parameters were kept the same for each measurement between comparing groups.

### Western blot

Proteins were first extracted from primary astrocyte cultures using RIPA buffer (50 mM Tris-HCl pH 7.4, 150 mM NaCl, 2 mM EDTA, 1 mM PMSF with 1% Igepal CA-630, 1% sodium deoxycholate, 1% Triton X-100 and protease inhibitor cocktail (1:100, Sigma P8340), followed by BCA protein assay (Thermo Fisher Scientific 23,225) to determine the concentration of each sample. 100 μg of solubilized proteins were boiled in Laemmli sample buffer for 5 min, subjected to sodium dodecyl sulfate (SDS)-polyacrylamide gel electrophoresis, and subsequently transferred onto nitrocellulose membranes. The blots were incubated with primary antibodies of anti-GFAP (Dako Z0334), anti-Fmrp (Abcam ab17722) and anti-GAPDH (Millipore MAB374) overnight at 4 °C, then with horseradish peroxidase-conjugated secondary antibodies for 2 h at room temperature. Protein bands were visualized with enhanced chemiluminescence reagents (Amersham Biosciences, Buckinghamshire, UK). The intensity of all resulting bands was quantified by densitometry using ImageJ, normalized to loading bands (GAPDH) and expressed as a percentage of WT controls. Triplicates were performed for each sample.

### Statistical analysis

The Student’s two-tailed *t*-test was performed to determine statistical differences between WT and *Fmr1* KO groups. Neuronal distributions of Cux1 and Ctip2 were analyzed with the one-way ANOVA (GraphPad 5.0), with Bonferroni’s correction for multiple testing. All images were blinded prior to analysis. Data were expressed as mean ± standard error of mean (SEM) and a significance level of *p* < 0.05 was used.

## Additional file

Additional file 1: Absence of FMRP in primary astrocytes derived from *Fmr1* KO mice. (PPT 585 kb)

## Data Availability

The datasets used and/or analysed during the current study are available from the corresponding author on reasonable request.

## Abbreviations

ASD: Autism Spectrum Disorder
CNS: Central Nervous System
FMR1: Fragile X Mental Retardation 1
FMRP: Fragile X Mental Retardation Protein
FXS: Fragile X Syndrome
GFAP: Glial Fibrillary Acidic Protein
KO: Knockout
MBP: Myelin Basic Protein
PV: Parvalbumin
WT: Wildtype
