# Supplementary material for: Altered cortical Cytoarchitecture in the Fmr1 knockout mouse
Source: Mol Brain. 2019 Jun 14;12:56. doi: 10.1186/s13041-019-0478-8 (PMC6570929; doi:10.1186/s13041-019-0478-8)
Supplement: Supplementary file 1 — Absence of FMRP in primary astrocytes derived from Fmr1 KO mice. (PPT 585 kb) [file 13041_2019_478_MOESM1_ESM.ppt]

## Slide 1
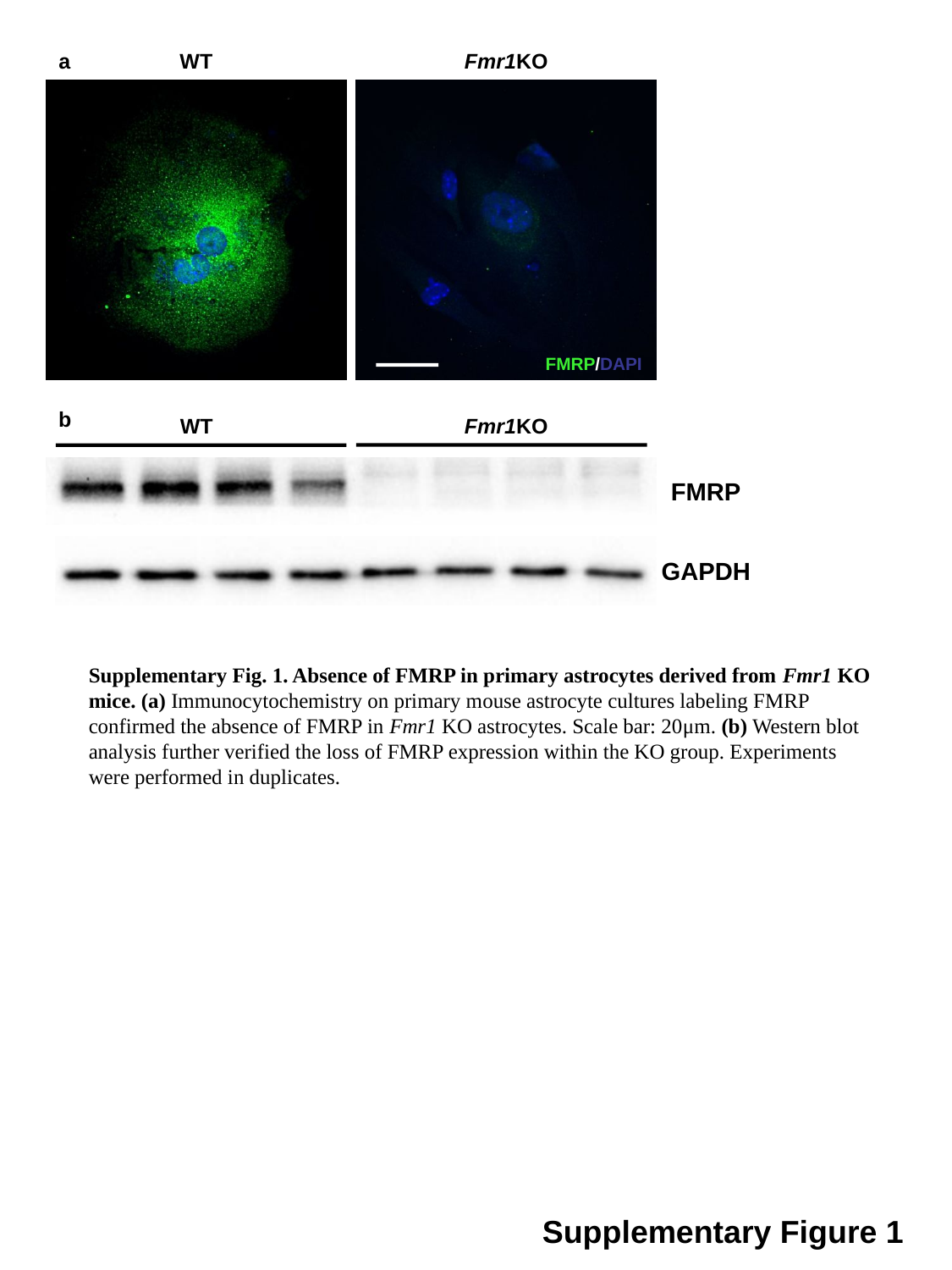

WT
Fmr1KO
a
FMRP/DAPI
b
WT
Fmr1KO
FMRP
GAPDH
Supplementary Fig. 1. Absence of FMRP in primary astrocytes derived from Fmr1 KO mice. (a) Immunocytochemistry on primary mouse astrocyte cultures labeling FMRP confirmed the absence of FMRP in Fmr1 KO astrocytes. Scale bar: 20μm. (b) Western blot analysis further verified the loss of FMRP expression within the KO group. Experiments were performed in duplicates.
Supplementary Figure 1
